# COVID-19 Pandemic—Frontline Experiences and Lessons Learned From a Tertiary Care Teaching Hospital at a Suburban Location of Southeastern India

**DOI:** 10.3389/fpubh.2021.673536

**Published:** 2021-06-11

**Authors:** V. Nirmal Coumare, Swati Jayant Pawar, P. S. Manoharan, R. Pajanivel, Lokesh Shanmugam, Hemanth Kumar, Abhijit V. Boratne, Balanehru Subramanian, Joshy M. Easow, B. Sivaprakash, R. Kalaivani, K. Renuka, S. Prabavathy, Kripa Angeline, Agieshkumar Balakrishna Pillai, S. R. Rao

**Affiliations:** ^1^Mahatma Gandhi Medical College and Research Institute, Sri Balaji Vidyapeeth (Deemed to Be University), Puducherry, India; ^2^Indira Gandhi Institute of Dental Sciences, Sri Balaji Vidyapeeth (Deemed to Be University), Puducherry, India; ^3^Central Inter-disciplinary Research Facility, Sri Balaji Vidyapeeth (Deemed to Be University), Puducherry, India; ^4^Kasturba Gandhi Nursing College, Sri Balaji Vidyapeeth (Deemed to Be University), Puducherry, India; ^5^Research, Innovation and Development, Sri Balaji Vidyapeeth (Deemed to Be University), Puducherry, India

**Keywords:** COVID-19, public health, health research service, health care setting, health care policies

## Abstract

The key challenges to any health care setup during emergency situations, such as that of the COVID-19 pandemic would be to rapidly address hospital preparedness and response tailored to the local population, societal influences, political factors within the existing infrastructure, and workforce. Second, to adopt and moderate policies, standard operating procedures (SOPs) and guidelines issued by national and international agencies, such as WHO, CDC, and the Indian Council for Medical Research (ICMR) were tailor-made to the local conditions of the hospital and community. In this publication, we have discussed the challenges and experiences in preparation and responses to the ongoing COVID-19 pandemic at a tertiary teaching hospital situated at a suburban locale in a small union territory. Puducherry is located in the South Eastern Coromandel Coast of India. The core processes, such as hospital preparedness, adoption, and amendments to SOPs based on dynamic changes in guidelines released by the central and local government, training given to health care workers, setting up the in-house diagnostic facility, surge capacity, management of supplies during the lockdown, infection prevention, and control and patient care are discussed. We have also reinforced our experiences in translating COVID-related opportunities for research and innovation in the form of awards and research proposals for the faculty and students of our institute. The lessons learned in terms of strength and limitations on the ground level of public health during this process is worth sharing as it would provide guidance in preparing the health care setups for pre- and post-pandemic.

## Highlights

- Every hospital, be it in an urban or a suburban location, needs to be prepared for any kind of unforeseen pandemic.
- A vigilant Hospital Emergency Committee consistently monitors the existing infection control practices and is also prepared for any pandemic situations, such as these.
- The Hospital Response Team makes and enforces “protocols” in line with the directions of the local and central government bodies.
- Preparedness of the hospital is essential in the transformation of the existing infrastructure and capacity for changing situations without affecting normal patient care.
- Financial contingency in the initial stage is only temporary. The returns at the latter stage would replenish the loss at the earlier stages.
- Hospitals should be kept abreast with various coping strategies of other health care setups and learn from shared experiences of others.

## Introduction

The COVID-19 pandemic continues to bring in new challenges every day to millions of people across the globe, in terms of providing health care, social obligation, economy, and mental health. Human coronavirus is known to humans earlier in various different forms, such as 229 E, OC43, NL63, HKU1, SARS-CoV, and MERS-CoV. The present form of novel coronavirus (COVID-19) originated from Wuhan, Hubei province of China, and spread to other parts of the world. Western pacific countries like Australia, New Zealand, Brunei Darussalam, Japan, and Singapore showed an early record of cases in January 2020 (1, 2). By the end of January 2020, the World Health Organization (WHO) declared COVID-19 as a “public emergency of International Concern” (3, 4). America and European and East Mediterranean countries by the end of February 2020 showed the first reports of COVID cases. Southeastern countries showed up the first report of cases from March 2020. Africa reported its first case by the end of March 2020. India was not among the worst-hit countries till May 2020, but by the end of 2020, the country ranked top in the number of cases in Asian countries (5). Lockdown efforts by the Indian government was appreciated by WHO and other countries as it “contained” the spread of infection in the initial stages. The efforts were mentioned as “robust and comprehensive,” although it may seem “aggressive.” Subsequent phases of lockdown did not show much impact on the containment of cases. With the second-highest population in the world, India, despite its rapid increase in numbers, is showing a very mild reduction of mortality (0.89%) and active cases (1.1%) as of October 2020 (5). India is a federal union comprising 28 states and eight union territories, for a total of 36 entities. Among all the Indian States, the largest and most well-developed states of this subcontinent—Maharashtra and Tamil Nadu—showed a rapid increase in the number of new cases, which was a matter of concern. This kind of surge is in spite of the availability of good hospital services from both the government and private sectors in these states. By October 2020, all the lockdown measures were lifted before the curve reached the plateau (6).

**Figure 1:**
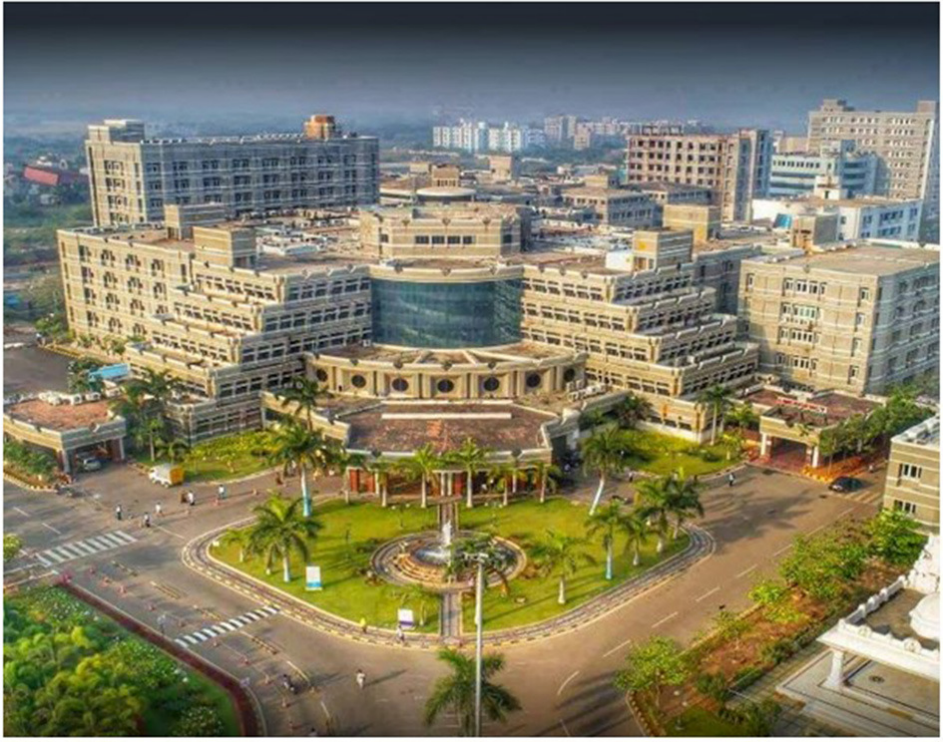
Geographic location of Puducherry and location of MGMCRI hospital in the outskirts of the small town Puducherry. Other affiliated Institutes of Sri Balaji Vidyapeeth–Sri Sathya Sai Medical College and Research Institute, Indira Gandhi Institute of Dental Sciences. Bed capacity 1,400, 15 modular operation theaters. Patient flow 1,000 out-patients 700 inpatients per day.

During the initial phase of the pandemic, many countries developed policies, standard operating procedures (SOPs), and guidelines at the national level [including the Indian Council for Medical Research (ICMR), Ministry of Health and Family Welfare, Government of India] (7) based on those issued by international public health agencies, such as WHO (8) and Centers for Disease Control and Prevention (CDC) (9) USA. Later, however, because of the rapid spread of COVID-19 in a shorter time frame and the lack of prior knowledge and available evidence on the risk of transmission, morbidity, and mortality (10), developers of guidelines were challenged to appropriate the SOPs suited to local and regional situations. Conflicting recommendations by various agencies, such as the case of wearing a mask between CDC and WHO, and a difference in opinion as to when it is safe to discharge COVID-19 cases from the hospital or end home isolation, etc. also added to the confusion. Given the dynamic nature of the problem and accumulated evidence over a period, the guidelines have been modified to ensure a reduction in the disease incidence. Therefore, the key challenges to any health care setup during emergencies, such as that of the COVID-19 pandemic would be to adopt and moderate various instructions issued by national and international agencies that are conflicting and changing from time to time to suit the needs of local community and resources. Figure 1 summarizes various factors and linkages to consider in preparing the health care setup before and during pandemic for adopting and preparing guidelines.

The Indian health care system is organized into primary, secondary, and tertiary levels. At the primary level are sub-centers and primary health centers (PHCs). At the secondary level, there are community health centers (CHCs) and smaller sub-district hospitals. Finally, the top level of public care provided by the government is the tertiary level, which consists of medical colleges and district/general hospitals. The number of PHCs, CHCs, sub-centers, and district hospitals has increased in the past 6 years, although not all of them are up to the standards set by Indian Public Health Standards (11).

Tertiary-level hospitals at medical colleges and district/general hospitals play a critical role in national and local responses to emergencies, such as communicable disease epidemics. These hospitals should be able to rapidly adopt various guidelines and address hospital preparedness and response for the pandemic tailored to the local population, societal influences, political factors within existing infrastructure, and workforce.

In this context, the present article describes frontline experiences and lessons learned from “Mahatma Gandhi Medical College and Research Institute” (MGMCRI), a tertiary care teaching hospital in the private sector in the suburban part of Southeastern India during the COVID-19 pandemic. MGMCRI is a teaching hospital and has the mandate of uninterrupted teaching and training to undergraduate, post-graduate, super-specialty students, and scholars. Furthermore, being a private teaching hospital does not receive any kind of external support in terms of finances even under pandemic-related emergencies for the treatment of patients. Despite being in a remote location, MGMCRI renders tertiary care in medical and dental services on a non-profit basis primarily to the rural population. The road, however, was never smooth and required continued attention to the safety of health care workers (HCWs) and doctors.

## Geographic And Ethnic Uniqueness

**Figure 2:**
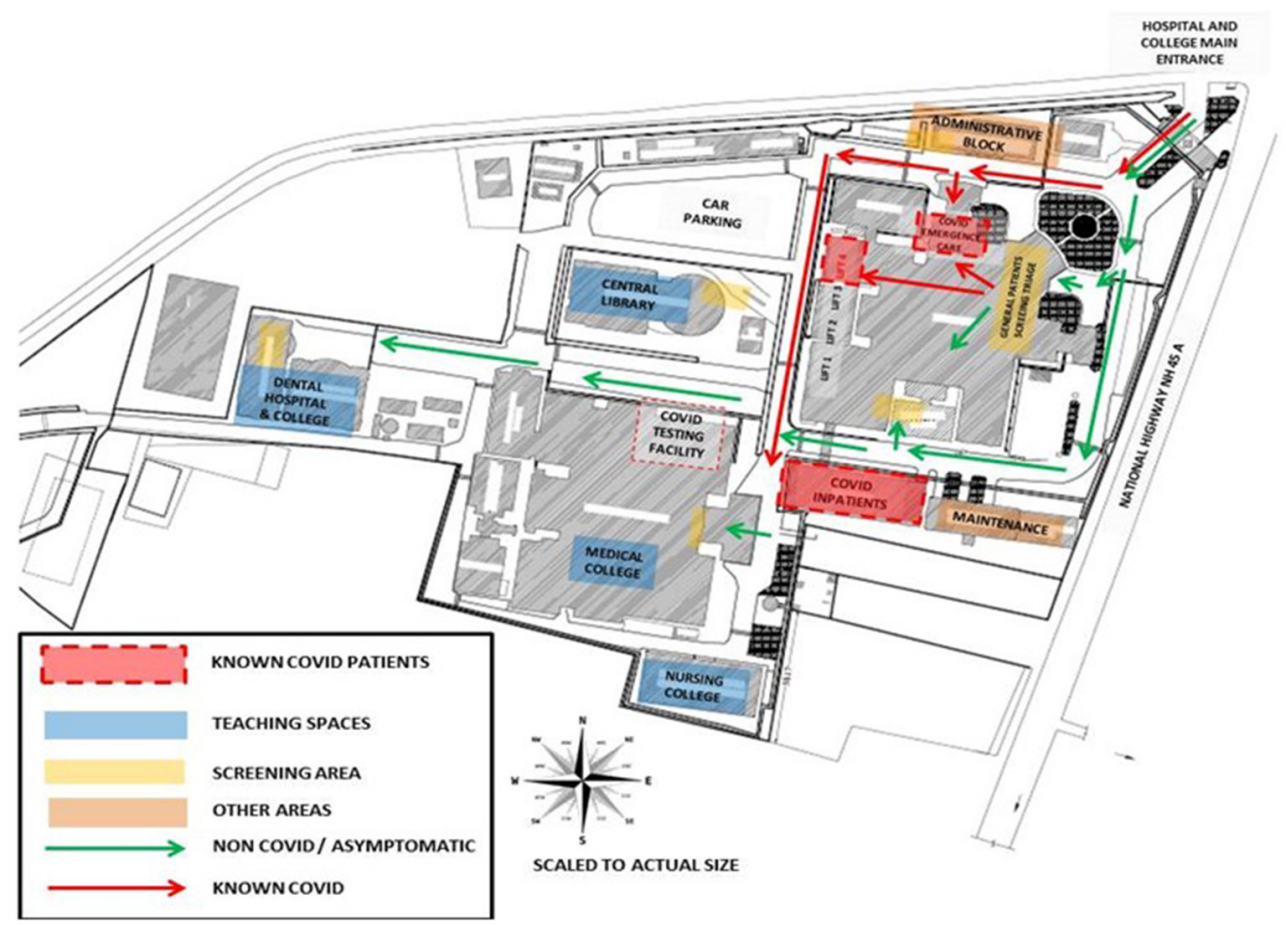
Hospital campus showing a planned identification of COVID care facilities, screening points, high risk zone, and other safe zones.

MGMCRI is situated in Puducherry (a union territory in the coastal stretch of Tamil Nadu—a south Indian State). Puducherry by itself is made up of pockets of land scattered in the region of Tamil Nadu. Geographically, the hospital is situated at Pillayarkuppam, which is a part of a suburban village commune Bahour, which administratively belongs to Puducherry (Figure 2).

Each country and region vary in terms of capacity, health policies of the government, and health infrastructure. Puducherry recorded 2500 COVID-affected cases per 1,000,000 of the population, which ranks second in India (12). Being the “*health indicator*” of the country, with the lowest infant mortality rate, it was an irony for us to witness these alarming statistics. In a small town with nine hospitals that cater for tertiary care, private multispecialty hospitals, 17 disease-specific clinics, four community health centers, 39 primary health centers, 77 sub-centers, 14 ESI dispensaries, and rural and urban health centers, it was saddening to note the increase of cases. Preventive and curative programs for tuberculosis, leprosy, filaria, malaria, and blindness control program with free cataract surgeries have curbed major illnesses in this town. Furthermore, Pondicherry had never witnessed a viral flu outbreak or a pandemic of this magnitude before. Arthropod-borne viral diseases like dengue have taken their toll on a few thousands of the population, with a few outbreaks in the last two decades. None of the hospitals in this region were prepared to handle the COVID disaster of such a magnitude.

With this background, the surge in COVID-19 infections can only be attributed mainly to the complacency and casual attitude of the public at large of this calm and peaceful town. In the initial phase, soon after Puducherry recorded its first case during the lockdown, the growth in a number of cases was well-contained. Pondicherry is a tourist destination for the neighboring states and also other countries, with its French legacy preserved in its French Quarter, with tree-lined streets, mustard-colored colonial villas, and chic boutiques and a seaside promenade running along the Bay of Bengal. Many tourists flood this small town on the weekends and vacations throughout the year. Therefore, the economy of this union territory depends on tourism. Lifting of lockdown and opening of borders to support the dwindling economy led to the surge of cases through migration of the “*floating population*.” As a result, the COVID pandemic further crippled the so-called “*health hub*” of the Indian subcontinent.

Hospitals play a critical role within the health system in providing essential medical care to the community, particularly during the pandemic crisis. As discussed above, the COVID-19 pandemic is teaching new lessons to health care providers and policymakers to adopt national and international guidelines and policies to develop “*tailor-made system*” solutions at the micro-economy level, yet providing effective treatment taking into consideration disease patterns in that region, the lifestyle of the particular population, food habits, socioeconomic status, apprehensions of the society, political factors, local administrators' policies, hospital infrastructure, workforce available, financial structure, and community.

With regard to many accreditation cycles from various quality councils, few hospitals have managed to establish disaster response teams and infection control protocols in place (13). MGMCRI was recognized as a COVID care center and a diagnostic center, by the local government after the first few months of the outbreak in this region. Only one other private hospital was given the testing facility for the entire region. This hospital provides its medical services to the villages in Puducherry and also to many villages and cities from the neighboring state—Tamil Nadu.

## Evolution as a Covid Care Center

The hospital had to go through various milestones in the evolutionary process of being a COVID care center, such as:

- Establishment of a Hospital Core COVID Committee (HCCC), which included members from clinical, human resources (HR), nursing, biomedical engineering, purchase, pharmacy, laundry, food and beverage, and transport departments. A contingency team of members was also formed.
- Preparation of SOPs for various processes adopting and moderating WHO/ICMR guidelines.
- Amendment of SOPs based on the dynamic change in the guidelines as and when released by the central and local government.
- Intensive training for health care providers.
- Setting up of an in-house COVID diagnostic facility.
- Logistics to scale up facility space for critical care, isolation, cohort, the accessibility of mechanical ventilators, and the availability of other resources.
- Wards to be reshuffled to accommodate COVID patients in an isolated facility.
- Respond to government directives in community service and provision of free beds to the government for COVID care.

A well-functioning hospital incident management system is essential for the effective management of emergency operations. As MGMCRI is already recognized by national and international accreditation bodies like the National Accreditation Board for Testing & Calibration Laboratories (NABL), NABH, and ISO, the disaster response team and some of the fundamental protocols of disaster management, infection control, and risk mitigation were already in place. The continuity of essential health services, e.g., emergency services, urgent surgical operations, and maternal and child care, was ensured during the outbreak season. Some of the important functions of various departments and individuals during the operation are summarized below:

**1. Internal and External Communication of the Facility**

The HCCC has devoted one nodal person from whom authorized information and the decisions taken by the HCCC on the safety of patients, visitors, doctors, and staff and students, circulars or guidelines arising from the government related to COVID would be conveyed to the hospital staff from time to time. The committee's role is to consider all SOPs made by the Health Ministries of local and national governments issued from time to time and moderate their relevance, take decisions to moderate to the hospital ecosystem, and supervise for compliance. All SOPs (Table 1) were carefully made and had information on the following:

- What functions does it affect?
- What are the procedures that are derived from this policy?
- Elements of the SOP workflow.
- Who does it affect?
- Who is responsible for its implementation?
- How is the SOP communicated to the stakeholders?
- Who needs to be educated on this SOP?
- Who will do the education?
- What is the media used for education?
- Who will monitor the SOP?
- How will the monitoring be done?
- When will the revision be done?
- Who has prepared this document? Contact details of the same.
- Who has approved this document?

**Table 1:** List of standard operating protocols and policies.

**S. No**.	**Name of the Policy/SOPs**
1	Policy for Contact Tracing of COVID19—Positive Employees of Sri Balaji Vidyapeeth, Main Campus
2	Policy for in Campus Contact Tracing of COVID19 (Version 1.2)
3	Policy for Contact Tracing of COVID19—Positive Employees of Sri Balaji Vidyapeeth, Main Campus (Version 3)
4	Policy for COVID19 Screening to Post-Graduate Courses 2020–2027
5	Policy for SBV Healthcare Workers Tested Positive for COVID19
6	SOP for Low Risk Quarantine
7	SOP for High Risk Quarantine
8	SOP for Setting up Isolation Facility/Ward
9	SOP for Dental Clinical Protocols
10	SOP for Rational use of PPEs (Version 1)
11	SOP for COVID19 Management
12	SOP for Preventive Measure to Contain of COVID19 in Skill or Entrepreneurship Training Institutions and Higher Educational Institutes

The SOPs were developed to handle COVID patients with mild, moderate, and severe symptoms. Flow charts were developed and circulated as SOPs to all health care providers for uniformity and also.to prevent litigations in this unfamiliar zone (3–5).

**Figure 3:**
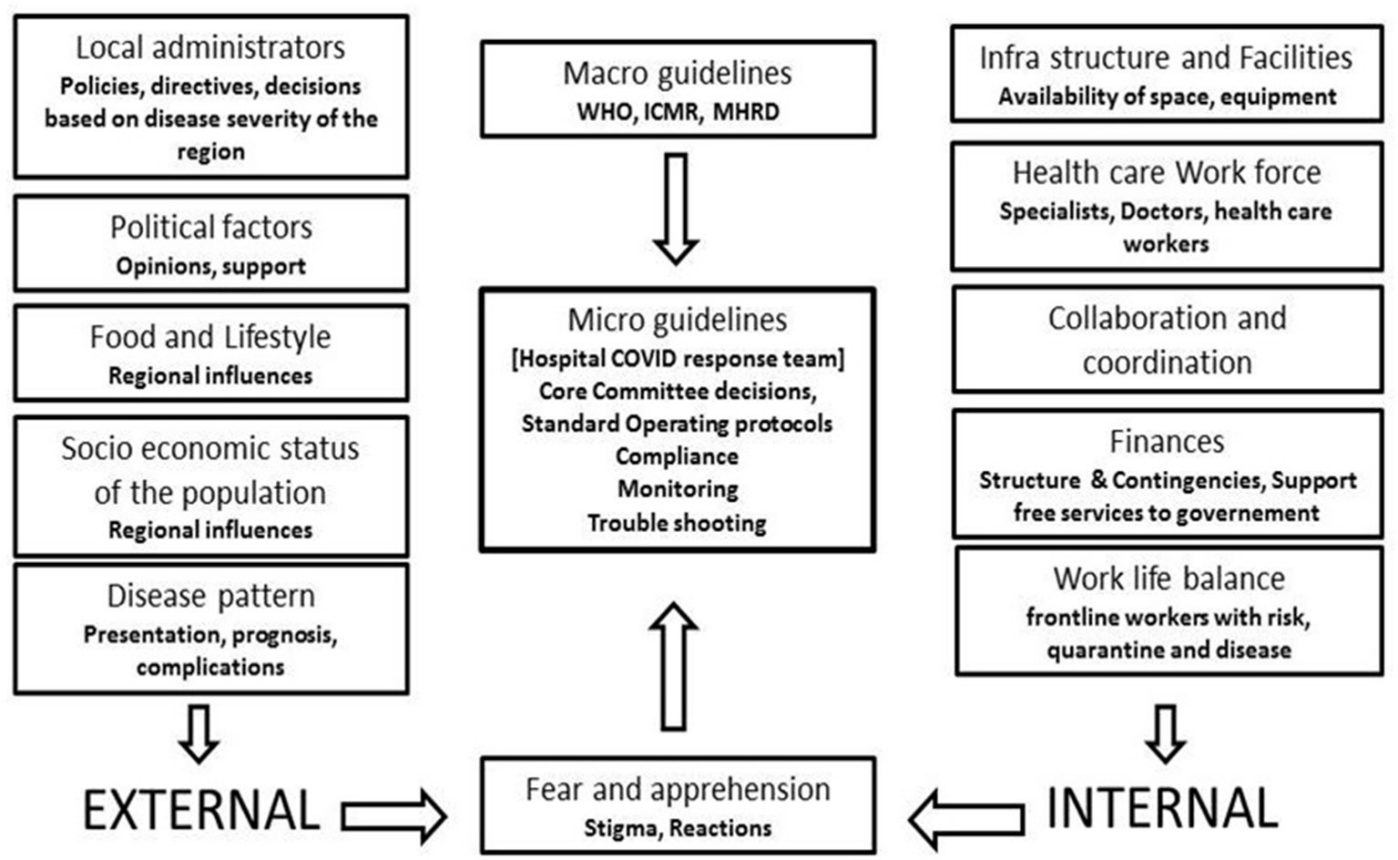
External and internal influences on development of micro guidelines which decides the preparation of standard operating protocols and other decisions of the response.

**Figure 4:**
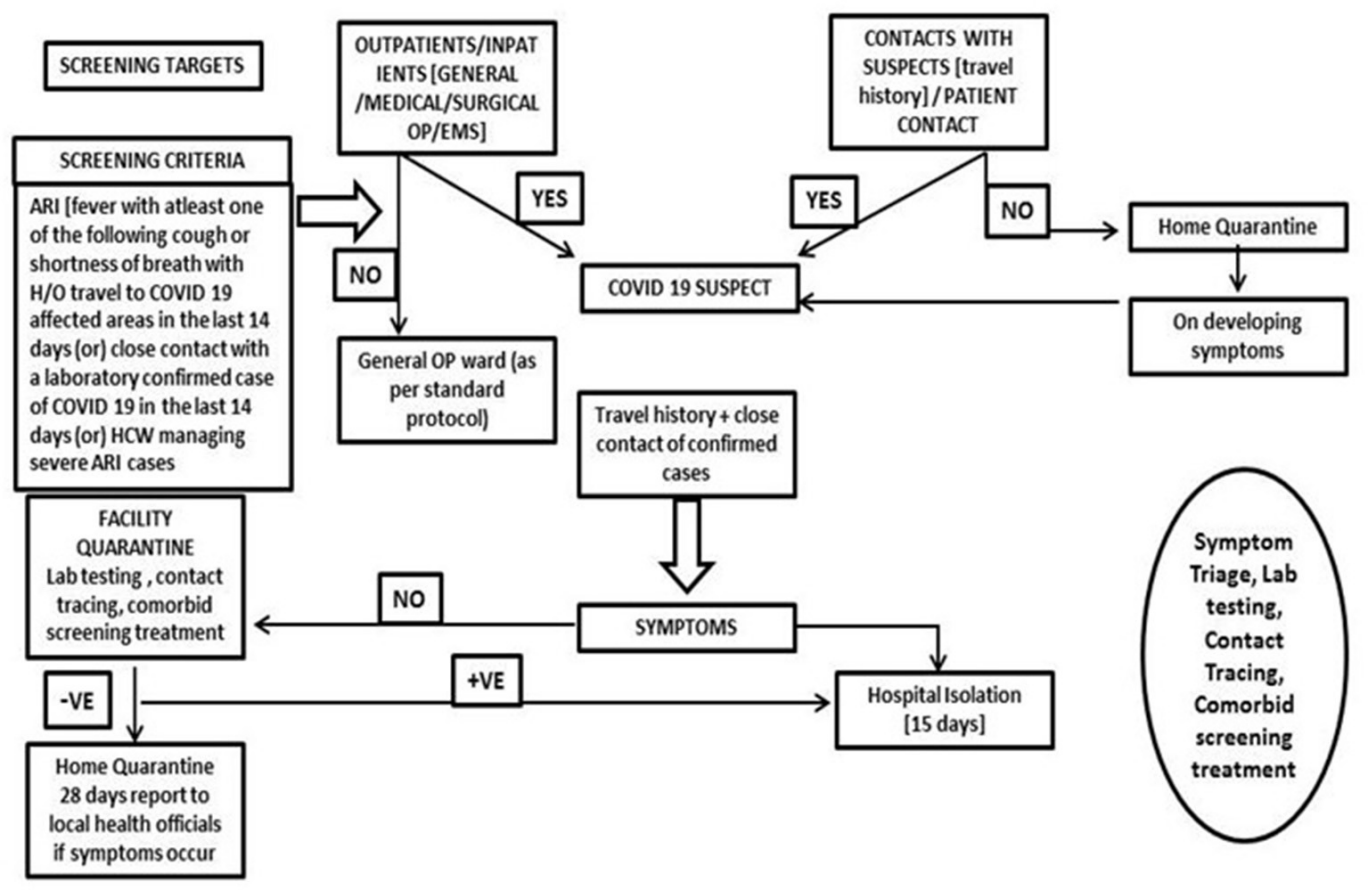
General patient screening protocol: screening criteria and triage policy.

**Figure 5:**
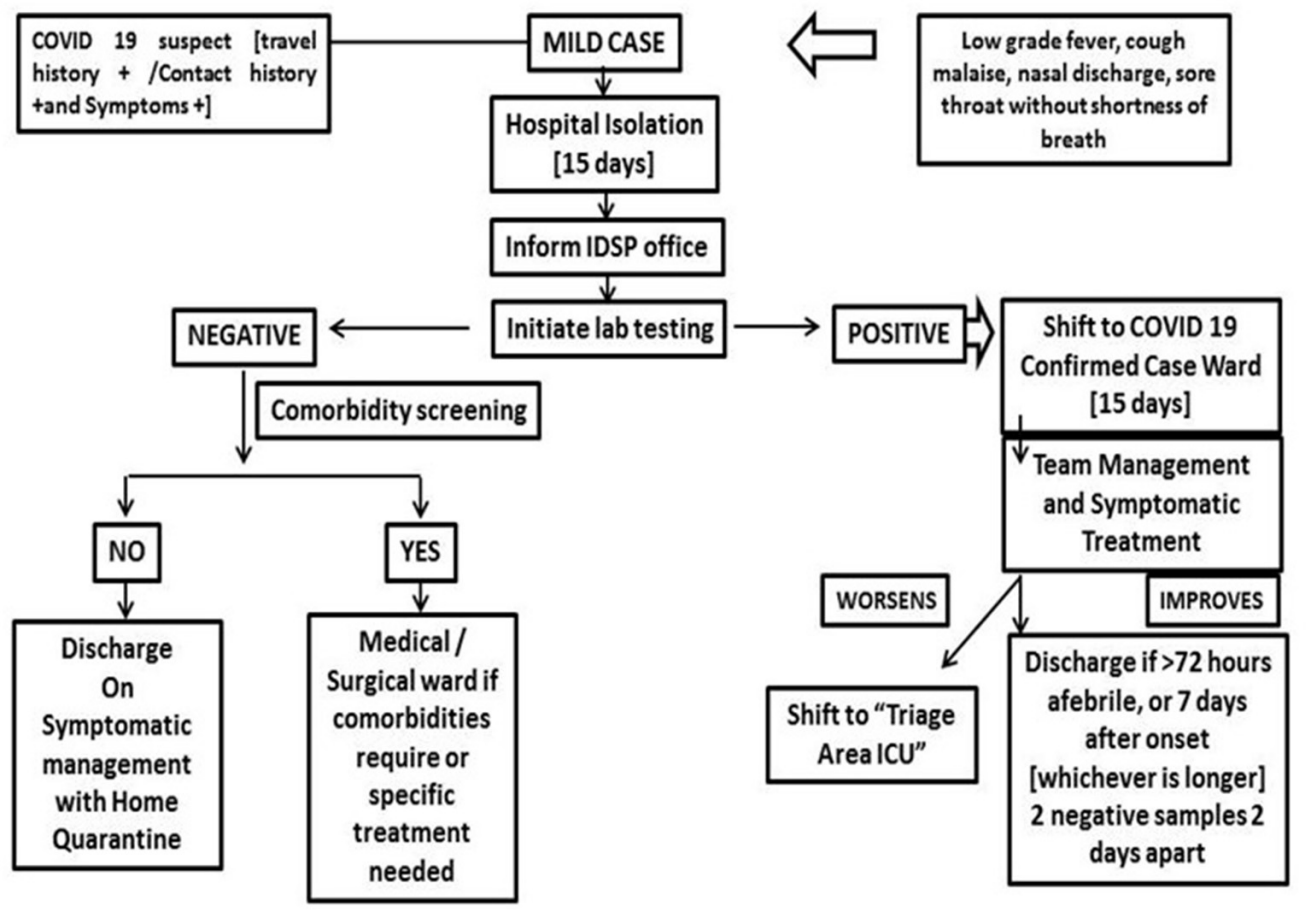
Patient management for COVID suspects (mild case) with presenting symptoms.

The hospital also designated a Public Relation Officer (PRO) to manage the flow of information from the hospital to the government/local authority, press, media, etc. All the information was passed on a regular basis to the Integrated Disease Surveillance Project (IDSP). IDSP is an online reporting system under the Integrated Disease Surveillance Programme (IDSP) of the National Health Mission Programme headquartered at Ministry of Health and Family Welfare Government of India. The key objective of this program is to strengthen/maintain a decentralized laboratory-based IT-enabled disease surveillance system for epidemic-prone diseases to monitor disease trends and to detect and respond to outbreaks in the early phase through trained rapid response teams (RRTs). The HR head of each hospital in the country is involved in data entry of real-time information in IDSP and, at the same time, communicating related information to the staff and the frontline workers regarding quarantine rules and facilities specially set up by the hospital for meeting the emergencies from time to time.

Continuous education and training for health care professionals and workers in the frontline was provided to provide quality care with extreme precautions. The residents and post-graduates involved in COVID care were educated on the policies, protocols, and guidelines from various statutory bodies and the hospital.

**2. Human Resource Activities**

In a dynamic and unpredictable situation like this pandemic, human resource management is a challenging task. Ensuring adequate trained staff capacity and continuity of operations in response to increased demand is important while maintaining the identified essential services. Furthermore, the HR department is also responsible in addressing liability, insurance, salary, management of leave application, and approval to the best of their capacity. Appreciably, HR has devised duty rotations in such a way that the demands are met throughout the period without overcrowding, as well as the arrangements for their transport, lodging, and food without putting anyone at high risk. As there is an increase in the demand for housekeeping and maintenance staff during these times, the rotation postings were planned round the clock and as is called 24 × 7.

**3. Tele-clinic Support**

To facilitate regular support to the patients during different phases of lockdown, MGMCRI set up a special Tele-clinic facility. “*Easy to understand advisories*” in English and Tamil (the native language of the region) developed in consultation with clinicians and the Community Medicine department are transmitted through short messaging service (SMS) to alleviate fear and apprehension and to provide practical advice on common medical problems. This facility was highly successful for uninterrupted communication. Education and training on the management of the Tele-clinic facility in the hospital were provided to health care professionals. It can be also told that this hospital is the first in this region to care for the public through teleconsultation.

The hospital also became a partner to the technology-enabled remote monitoring and counseling platform for home-isolated COVID patients, which was also launched by the local government in partnership with Step One organization (the largest doctor volunteer group to fight COVID). The platform is based on the involvement of doctors and tele-counseling by nurses, psychologists, and social workers with sufficient training provided by the Step One organization. The purpose is to reduce the burden of public hospitals to treat high-risk cases and to optimize the health care workforce. The platform also aims to improve the physical and mental well-being of COVID patients quarantined at home. Faculty members and post-graduate students of Kasturba Gandhi Nursing College volunteered to participate in this platform and played an important role as counselors and provided counseling services. Tele-counseling was done through Fresh Desk Mobile App with services, such as telecommunication with the allotted home-isolated clients; daily identification of COVID symptoms and referral to hospitals; identifying the common psychological problems and providing counseling to the needed clients; and providing psychological support to home-isolated clients and explaining the do's and don'ts during pandemic.

**4. Surge Capacity**

Surge capacity is the ability to expand health services beyond their normal capacity to meet the increased demand for clinical care. COVID-19 cases increased rapidly, and there was an increase in demand for patient care and admissions over a prolonged period of time. Calculation of “*maximal case admission capacity*” is determined not only by the total number of beds but also by the availability of human resources, the adaptability of facility space for critical care, isolation, cohort, the accessibility of mechanical ventilators, and the availability of other resources. The most important concern is having a medical space to create a new facility with reduced or no risk of COVID transmission. In other words, it meant creating a facility wherein HCWs are not at risk and the patients are attended to with caution. In this aspect, many of the tertiary care hospitals in Europe, Australia, and Asia (14–17) have published their experiences. An additional investment was recommended to the Kenyan government to enhance the surge capacity in terms of ICU and ventilated beds in hospitals (18). The triage system of evaluation and categorization of the patients was required for optimal utilization of the resources for medical care of everyone at once. Thus, an efficient and accurate triage system and an in-patient management strategy were organized to ensure adequate treatment of COVID-19 acute respiratory infection. Based on the previous experience on MERS in 2015 in South Korea, health care facility-associated nosocomial route of infection was considered a potential source for COVID-19 transmission (19, 20). To address this, MGMCRI developed its own SOPs (Figures 1, 3, 4) of triaging patients by establishing flu clinics at the entrance and taking in the patients based on their symptoms, travel history, etc. So, the hospital earmarked various locations for mild to moderate cases, critically ill cases, and patients needing surgical/cath-lab procedures or deliveries and other asymptomatic patients coming from non-containment zones.

Due to the infectious nature of SARS-CoV-2, the number of patients with other regular ailments in both inpatient and outpatient wards has reduced drastically since the first week of April. This prompted us to shut down one floor completely and keep others operational for regular patients. Beds for various purposes, such as pre- and post-operative surgical care and general medicine and allied specialties were earmarked beds. So, a decision to escalate or de-escalate depended on the utilization of beds.

There was speculation that there could be a surge of patients after the lockdown was lifted; this is where there was a need to prepare for a surge to manage not just for the COVID cases but also for regular non-COVID patients who were unable to reach the hospital because of the lockdown. A very significant point is to adapt admission and discharge criteria and prioritize patients and clinical interventions according to available treatment capacity on demand.

Initial phases of lockdown had a total shutdown of all elective procedures in ophthalmology, otolaryngology, and dermatology. By April 2020, all procedures resumed including adequate infection control protocol and other safety precautions. The emergency ward took care of COVID patients who developed acute complications. The existing emergency ward was shifted to another place. An “*exclusive facility*” with 200 beds was created in the campus premises isolated from the other areas, by “*rearrangement of various facilities*” like the Center for Music Therapy Education and Research (CMTER), the Center for Yoga Therapy Education and Research (YTER), Internal Quality Assurance Cell (IQAC), Medical Informatics, and wards of dermatology and venereology, surgery, and ophthalmology. A 102-bed facility was also assigned in the medical hospital block for COVID patients, of which 20 beds were assigned to in-house employees who succumbed to the infection. Ten critical care beds and intensive care units with ventilator support system were dedicated to COVID-related emergencies. The general wards also accommodated patients with mild to moderate symptoms that were referred from government hospitals. Apart from the general ward, COVID care special wards were also made available. During the late phase, the hospital assigned 25% of its total capacity of 1400 beds to COVID care. The hospital has also prepared a plan to expand the bed capacity if the need arises. MGMCRI of Sri Balaji Vidyapeeth University was also recognized as one of the two COVID testing centers among the seven private hospitals at Puducherry from the month of June 2020.

**5. Logistics and Management of Supplies, Including Pharmacy Supplies**

In the initial phases, there was a shortage of supplies either due to non-availability with the vendors or because transport was banned and borders were sealed. Personal protective equipment (PPE), N95 masks, and sterilant for washing hands are crucial for protecting frontline HCWs who were the worst hit. In consultation with basic virology laboratory, infectious disease experts, school of pharmacy and pharmacologists, and purchase and store managers, makeshift arrangements were made. PPE was stitched by tailors using impermeable wrapping material used for procedure trays with quality checks and supplied. N95 masks were reused using appropriate protocols particularly UV treatment of the masks with specific dosing protocols and full surface area illumination to ensure proper inactivation of viral particles with minimal mask degradation (21). Sanitizers were prepared in-house by the Department of Pharmacology using standard formulations. These alternatives with the other innate measures for non-crucial procedures aided to cope up with temporary shortages in the hospital. After the lift of the ban on travel and transport across borders, the issue pertaining to the availability of essential supplies was resolved. The purchase department in MGMCRI has ensured an uninterrupted supply of demanded items, following all the quality checks. We ensured adequate PPE (i.e., medical/surgical masks, N95/FFP2 respirators, gloves, gowns, and eye protection), which were made easily accessible to staff.

**6. Essential Support Services**

During the lockdown of 3 months, the services related to food and beverages (F&B), transport, laundry, medical gases, and information technology were severely jeopardized. F&B services, in general, have been outsourced for supplies in the hospital as well as for the staff, students, and visitors; transport and supply of vegetables, breads, eggs, etc. were severely affected. However, the experienced hospital personnel manager with his team effectively managed issues related to essential support services. This was possible because of linkages with local police and trustworthy vendors and transporters. The doctors on duty were provided with food prepared in the hospital kitchen on a regular basis.

The hospital has an automated laundry wing for an estimated capacity. The frequent need for a change of linen and other materials overloaded the laundry wing. Since the capacity was fixed, it was made to run overtime to manage the surge of work.

Many employees and faculty employed in the hospital travel every day from neighborhood districts outside the union territory with lockdown orders, so it was not easy for them to commute on a regular basis. Appropriate permissions were obtained and identification stickers for personal vehicles were arranged. Misuse of such permissions was also monitored. A considerable number use the hospital bus services. The challenge for the transport department of the hospital was to provide uninterrupted service with only 50% capacity in all vehicles following thorough disinfection protocols. Although the infection incidence has come down in recent times, this practice is continued with a sizable financial burden on the transport budget.

The information technology (IT) department in the hospital took no time to gear up with increased demands due to the pandemic. Since the patients are in isolation wards, the devices were installed in the wards for communication between the admitted patients and their attendants and visitors. For making payments to various hospital services, the Credit option was enabled for patients facilitating delayed payments. The IT-enabled Tele-clinic and mind clinic services support includes troubleshooting.

**7. Infection Prevention and Control**

The hospital is well-experienced in infection control. The Hospital Infection Control Committee (HICC) headed by a microbiologist plays an important role in infection prevention, surveillance, and training. The HICC besides specialized infection prevention, surveillance, training activities, rational use of PPE—a precious commodity and special needs of the biomedical waste management in laboratory and ward area was managed. The intervention was targeted to minimize the risk of transmission of health care-associated infection to patients, hospital staff, and visitors.

All entry points in the hospital, college, library, and residential areas have screening points. It was ensured that HCWs, patients, and visitors are aware of respiratory and hand hygiene and prevention of health care-associated infections and physical distancing to avoid contact and droplet transmission.

Verbal instructions are provided regularly apart from informational posters and cards, which were displayed in strategic areas. It was strictly monitored that all HCWs are applying standard precautions for all patients. Precautions on potential infection due to aerosol droplets during coughing and sneezing and physical contact are recommended for suspected or confirmed COVID-19 patients. These precautions were instructed to be continued until the patient is asymptomatic. It was ensured that all instruments used are either single use/disposable. Equipment (e.g., stethoscopes, blood pressure cuffs, thermometers, and food trays) that need to be shared among patients were instructed to be cleaned and disinfected between use for each patient with surface disinfectants. Implemented methods of routine cleaning and disinfection of ambulances follow the recommended standards and guidelines for COVID-19. A cleaning protocol for the Institute buses that ferry employees daily wasalso implemented.

The training was given to staff on standard, contact, droplets, and airborne precautions (including correct use of PPE, donning and doffing, masks tested for fitting, hand hygiene, respiratory hygiene, etc.). The HCCC has circulated the reuse policy for N95 respirators, which has considerably brought down the usage and purchase of this commodity. It is worth mentioning that two of our infection control nurses who were certified Yellow belts in the Lean Six Sigma supervised the use of PPE and other infection control protocol compliance via their constant rounds and vigilance. In general, moving and transporting patients out of their room or general ward is avoided unless medically necessary. Designated portable X-ray equipment and/or other designated diagnostic equipment have been used all the time. If transport is required, predetermined transport routes are used to minimize exposure to staff, other patients, and visitors, and the patient has to use a medical mask if tolerable or respiratory hygiene is reinforced. Also, it was ensured that HCWs who are transporting patients perform hand hygiene and wear appropriate PPE.

The HICC prepared protocols for rational use of PPE based on the categorization of risk groups into high, moderate, and low risk based on the exposure to potential asymptomatic patients. For strict compliance, supervisors were assigned to do the job. An SOP for use of PPE in non-COVID areas was also prepared for cost-saving purposes. Outpatient and inpatient areas were identified along with their activities and responsibilities to classify them as per the above risk groups, and the use of PPE, N95 masks, protective glasses, and face shields was as accordingly recommended. Use of PPE for non-COVID patients—obstetrics and gynecology surgical patients, dialysis units, operation theaters, emergency services, and other supportive services including ambulance services—was also defined based on the aforementioned criteria and risk categories. PPE use is made mandatory for treating confirmed or suspected cases of COVID in all areas including testing centers. Specially designated lifts took care of COVID patient transport to special wards in designated isolated areas, in carefully planned exclusive circuits in a manner that is not frequented by others to avoid contamination. Pathways connecting staircases from known COVID to non-COVID areas were sealed. Visitors were restricted to those only who are found essential for patient support.

The hospital with its risk mitigation system and well-outlined protocols is supervised from time to time to take care of in-house infection spread among the workforce, residents, and patients. The contact tracing committee from the Community Medicine department took care of tracing the source of infection and possibilities of its spread among faculty, employees, and post-graduates. The tracing committee followed an SOP for the same, which included the personal details and retrospective details of the contacts, categorizing them as high, moderate, or low risk along with other presenting symptoms. Quarantine orders were tailor-made for the aforementioned risk groups, and compliance to the protocol was monitored for employees and students. Movement restriction orders and Quarantine release orders are given to people who are categorized in the risk groups. About, 89 HCWs turned out positive, of whom 41 were doctors, 36 were nurses, and 12 were other staff. Nevertheless, it was found through contact tracing that none of these HCWs contracted COVID-19 in the hospital through direct patient contact while on duty, indicating SOPs for infection control implemented are effective.

**8. Patient Care**

**Figure 6:**
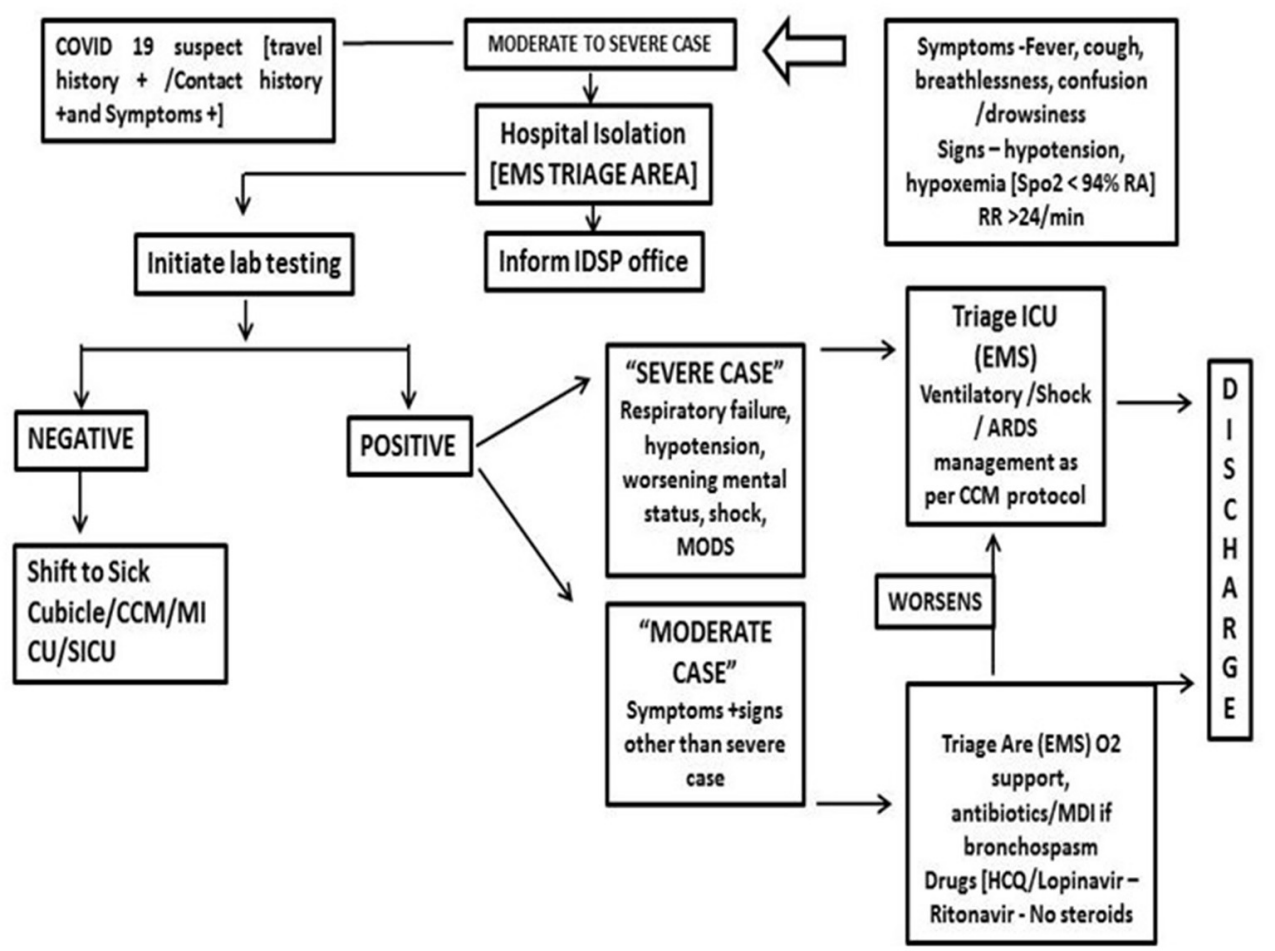
Patient management for COVID suspects (moderate to severe case) with presenting symptoms (IDSP, integrated disease surveillance project; CCM, critical care medicine; MICU, medical intensive care unit; SICI, surgical intensive care unit; EMS, emergency medical services).

The triage system was used to determine which groups of the patients should receive treatment and care services based on their clinical status, the prognosis of the disease, and available resources. To ensure adequate treatment of COVID-19 acute respiratory infection, an efficient and accurate triage system and an organized in-patient management strategy were implemented (Figure 6).

All the entry points are planned with a “*patient triage*,” which monitors and categorizes patients by recording temperature and recording of symptoms and cursory examination at safe distance. SOPs were prepared for asymptomatic, mildly symptomatic, and severely symptomatic patients, and they are carefully mobilized to designated areas. As mentioned before, separate isolation wards were allotted for mild to moderate suspected cases and confirmed cases were accommodated in the dormitory. Critical care setup was augmented for confirmed and severely ill cases. Isolated labor room and operation theater (OT) procedures with negative pressure were set up by applying exhausts. One critical care facility was converted to a COVID ICU to manage COVID emergency cases. All the wards where COVID patients were kept had a standalone air-conditioner facility.

Of the 1,000 patients treated for COVID-19, 24 patients died due to cardiac arrest and two patients recovered with critical care and the remaining patients recovered eventfully over a period. The hospital with its specialists in critical care, anesthesiologists, pulmonology, and general medicine provided round-the-clock services on rotation duty with quarantine orders. Extracorporeal membrane oxygenation (ECMO) facility, which was not available in this hospital, was a limitation in providing very advanced care. As no other neighboring hospital is equipped with such a facility, patients requiring such support would be referred to another tertiary care center of the adjacent city.

We ensured that all staff were aware of the national and international guidelines (WHO and ICMR) for case management. The guidelines included case definitions for suspected/probable/confirmed cases, common clinical symptoms associated with COVID-19, indications for admissions, recommendations for testing, management of cases based on severity, discharge criteria, and recommendation on drug therapy. So far, more than 1,000 COVID patients were given the care with more than 99% to complete recovery. Common complications observed were arrhythmias and cardiac arrest. The discharge policy was also based on a “*reverse triage*” system where priority on patient discharges was planned.

**9. Community Surveillance and Reaching out to the Society**

**Figure 7:**
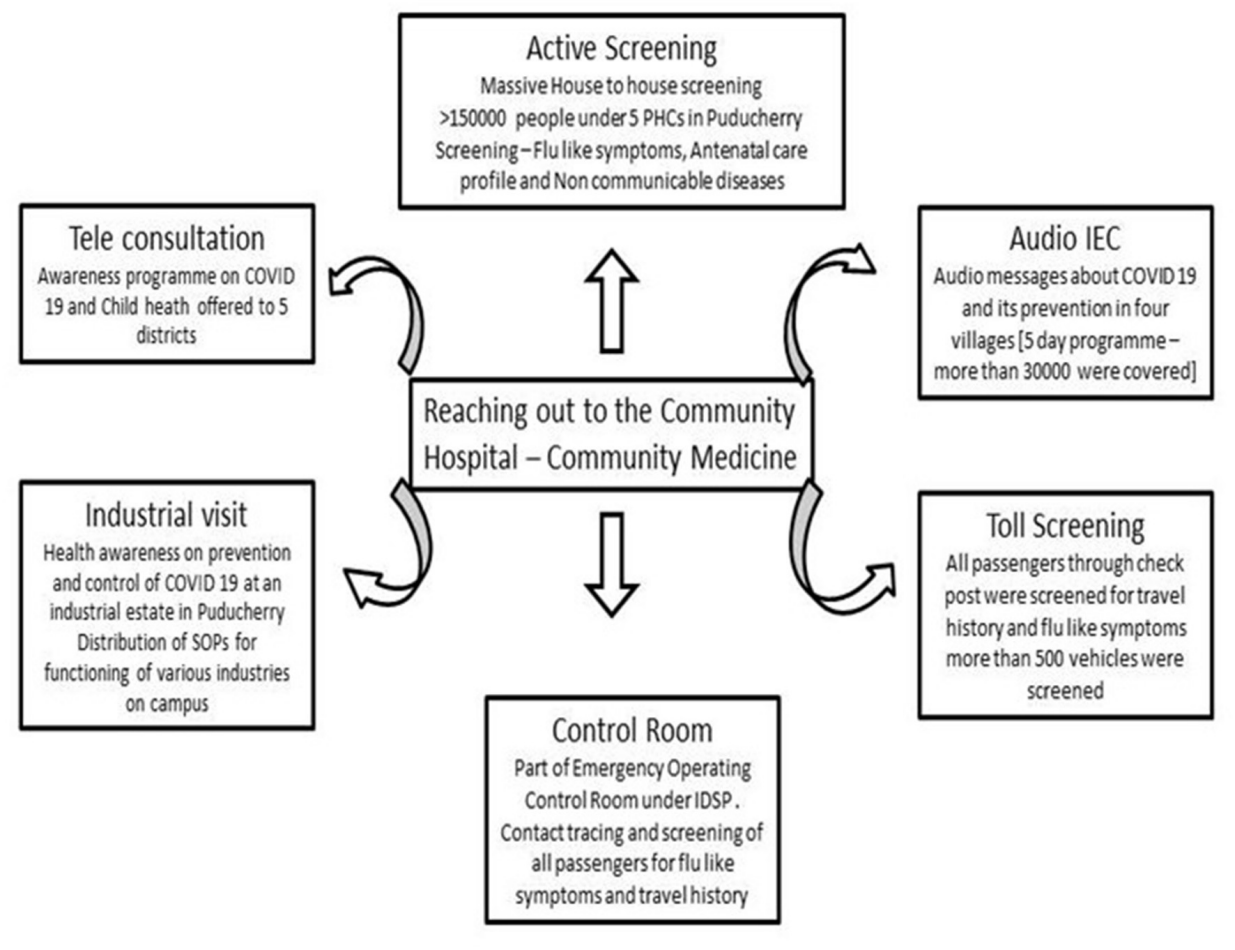
Depiction of community services to Puducherry and neighboring places.

Recognizing and immediately reporting unusual health events (e.g., clusters of cases, atypical clinical presentations, etc.) occurring in health care facilities are the cornerstone of the early warning function. This task of early warning function by HCWs, along with the laboratory and epidemiological data obtained through systematic collection and analysis, allows the public health authorities (Director of IDSP) to monitor the progression of COVID-19 and inform interventions for those at the highest risk of the severe outcome and helps hospital managers to plan accordingly. One check post near the state border was allotted to the hospital by the state authorities to screen the national and international passengers crossing daily for symptoms of COVID-19. Information Education and Communication (IEC) materials were prepared, which were disseminated to four suburban regions of Puducherry. The hospital was allotted four primary health centers by the Ministry of Health and Family Welfare of the Government of Puducherry for a door-to-door survey program. Mobile app data collection was done for more than 80,000 people in a span of 45 days. The post-graduates of a government hospital of the neighboring state were also involved in supporting the contact tracing activities. Tele-consultation for five districts was done for patients seeking answers to COVID-related queries. These activities were undertaken by the hospital Community Medicine department jointly with the Health Department Government of Puducherry (Figure 7).

**10. Laboratory Services**

Laboratory services are most essential for the diagnosis and clinical management of both COVID and non-COVID patients, as well as for the hospital-based surveillance of COVID-19. The hospital has a well-equipped research laboratory (Central Interdisciplinary Research Facility), certified by the Scientific and Industrial Research Organization of the Department of Science and Industrial Research (DSIR) of the Government of India. Besides, as in every hospital, there is a Central Diagnosis Laboratory for testing parameters, such as complete blood count, biochemistry profile, electrolytes, blood gas analysis, blood culture, sputum examination, etc. However, to address COVID testing through RT-PCR, a special BSL2 laboratory accredited by NABL is mandatory to be recognized as a testing facility. Since we have had a Molecular virology lab in place with Bio Safety Laboratory (BSL-2 and BSL-3) facility in the hospital premises, we could easily manage the COVID-testing process with the available facility and expertise. In order to establish as many COVID testing laboratories as possible in the country, the Government of India desired assessors of the NABL accreditation system to fast track the process. Accordingly, the hospital premises were inspected for testing methods and SOPs through a video conference drill for 8 h starting in the evening till midnight before according accreditation certificate. NABL is an accreditation body of India and had been set up under Mutual Recognition Arrangements (MRA) signatory to International Laboratory Accreditation Cooperation (ILAC) as well as Asia Pacific Accreditation Cooperation (APAC) for the accreditation of Testing and Calibration Laboratories (ISO/IEC 17025). As of November 20, 2020, India had 1150 RT-PCR for COVID-19 in the public sector and 634 in the private sector from only 35 in March 2020.

Altogether, the hospital established a laboratory referral pathway for the sample collection, identification, confirmation, and monitoring of COVID-19. The staff has been trained on packaging and transportation procedures for specimen referrals in accordance with national and international transport regulations and requirements.

**11. The well-being of Health Care Professionals and Workers**

Well-being is the experience of health, particularly good mental health, a sense of meaning or purpose, and the ability to manage stress. Crises like these can damage the well-being of health care professionals and workers. The mission of the battle against COVID toward service and rehabilitation of society was well informed to the frontline workforce. Infrastructure and amenities for protection and health promotion were made available. The entire workforce that was on duty was provided with food and hospitality with proper hygiene precautions and infection control protocols. Many health care professions reach the hospital through public transport or private mode of conveyance crossing the borders of the union territory into the next state. Feedback from the employees was taken to address the difficulties they face in times of crisis. A team was set to analyze the feedback with necessary follow-up action. The Department of Psychiatry addressed stress and other mental health issues related to the pandemic by offering “*Mind Clinic*” —a free consultation facility with hotline numbers made accessible to faculty and students of the institute apart from its primary purpose of addressing the public.

**12. COVID Related Research Opportunities**

Alongside responding to the pandemic, COVID/general patient care, and teaching, the scope on research and innovations has also been explored. The office of the Vice president of Research, Innovation and Development has announced a “*call for innovative ideas to address unique challenges of Indian health care sectors for management of COVID-19*” for cash prizes. The Chancellor of the University encouraged potential areas of research to be identified with regard to COVID for a sumptuous grant of 20 million INR (~280,000 USD). As we communicate this report, the hospital was given responsibility by the Central Drug Licensing Authority to conduct phase 3 clinical trials of randomized, double-blind, placebo-controlled, and multicenter study to evaluate the efficacy, safety, immunogenicity, and lot-to-lot consistency of indigenously developed whole virion inactivated SARS-COV-2 vaccine in 1,000 adults ≥18 years of age. The clinical trial is ongoing as per targets.

## Problems, Challenges, Opportunities To Strength

**Figure 8:**
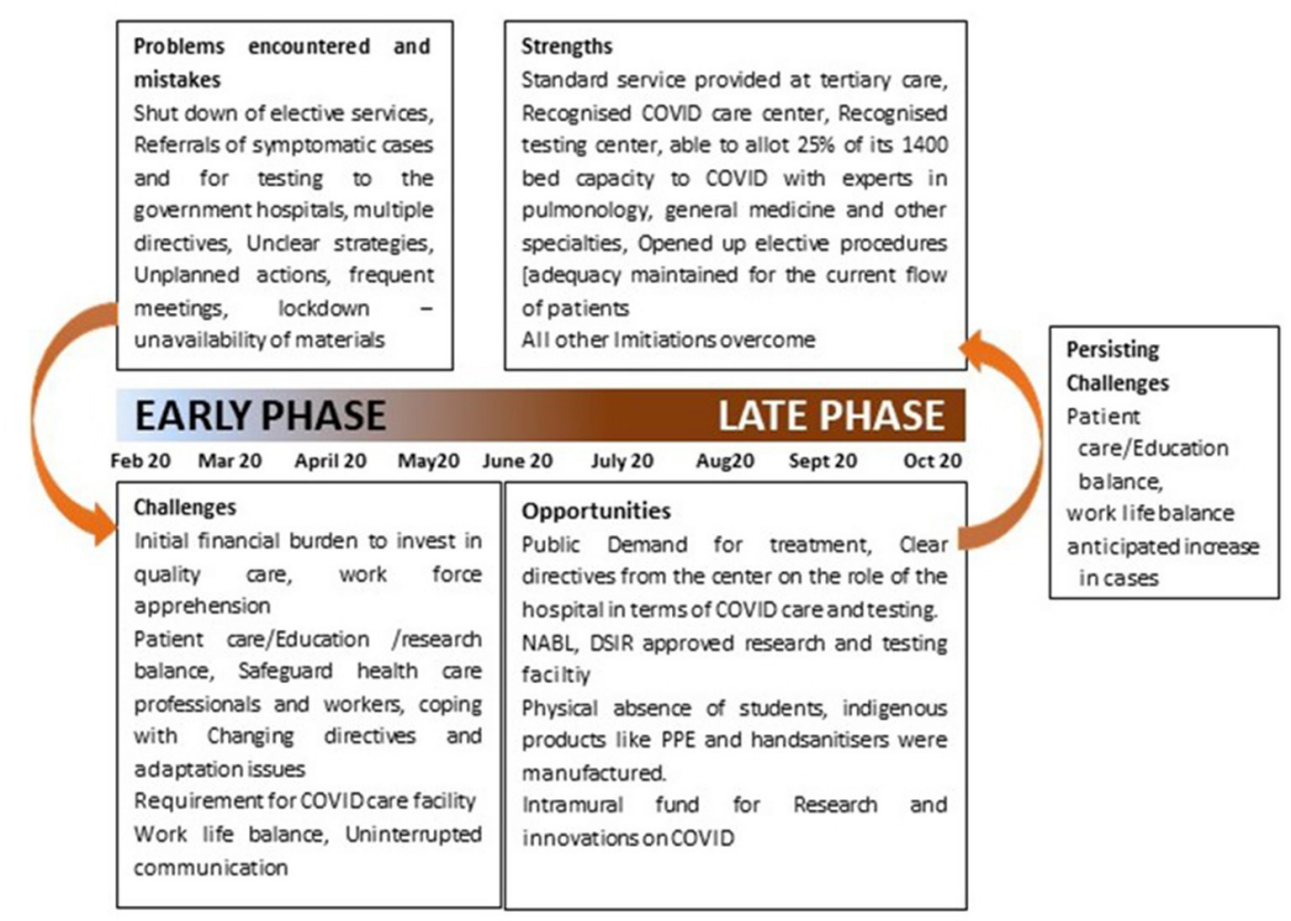
Depiction of various problems faced, opportunities seized which was used to convert the challenges faced to strengths.

During the pandemic and due to lockdown imposed in the country, most of the small, medium, and large hospitals had a reduced inflow of patients and the inpatient admissions have come down from 70% to about 10–20% or maybe less in some cases. OPDs were either closed or reduced (22). So, managing hospitals in times of high risk of infection and an appalling dip in the revenue became the biggest challenge faced by hospital owners, directors, superintendents, chief executive officers, and managers (Figure 8).

## Lessons Learned

**Figure 9:**
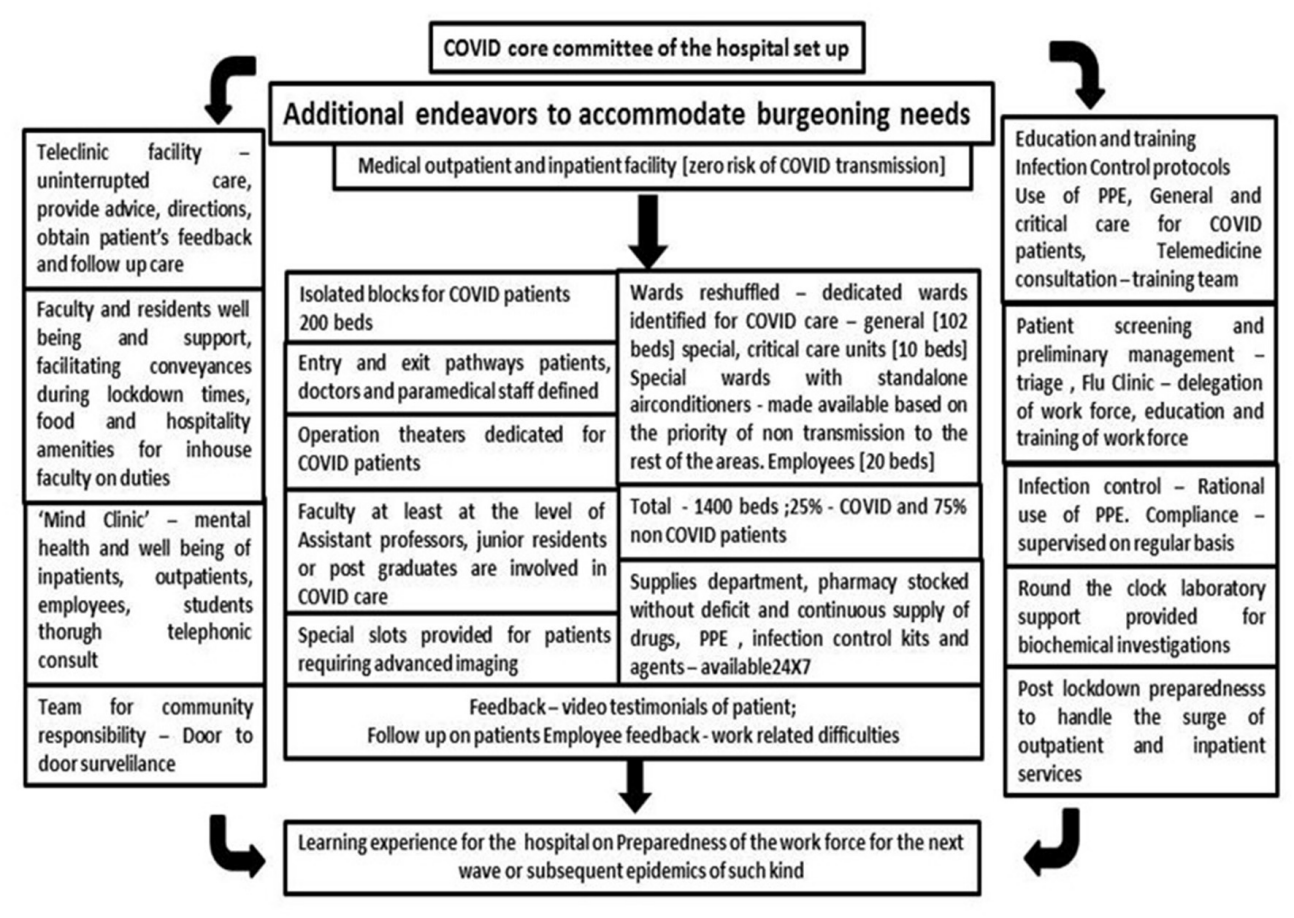
Master work flow of coordinated response from the hospital toward COVID management.

- Establishment of an HCCC is necessary with its regular meetings as the response team played a key role in the development of standard operating protocols (SOPs) tailor-made to local needs adopting and moderating the changing directives from the health authorities at national and international levels. This experience is unique and requires more documentation considering external and internal influences that permit designing SOPs that are location-specific and implementable in resource-scarce conditions (Figure 9).
- We learned from our experiences that integration of various resources and their coordination in terms of the cohesive workforce with an effective health care team and a response team for disaster management, flexible hospital infrastructure, state-of-the-art equipment, continuous availability of drugs and other supplies, and transport and maintenance departments are needed.
- The frontline health care professionals and workers need continuous protection to contain the infection among the workforce.
- The fear, anxiety, and apprehension among the faculty, residents, and patients need to be alleviated to promote mental health and well-being, using timely assurance and counsel by a team. Well-being can be also through care and concern, quarantine facilities, transport facilities, food, and hospitality during the duty hours.
- A trans-disciplinary team approach may be needed in such a crisis, whenever the need arises.
- Contingency in terms of financial resources, manpower (health care professionals, paramedical staff), and space availability would help to answer the needs for surge capacity. This was the toughest challenge as there was no external support. This hospital provided workforce to handle the admission and COVID patients' care in other government hospitals of this region.
- Optimizing the workforce and uninterrupted supplies of commodities for quality care were a challenge to be faced and require hospital preparedness through a response team.
- Hospitals should not only rise to the occasion for management but also assume a social responsibility of health education among the public on good practices of healthy life and good nutrition as a preventive measure in inpatient care.
- A smart leadership with good teamwork in the response team is essential to manage the workforce entrusted with COVID patient care in the frontline.
- Above all, we realized the importance of the Information and Communication Technology (ICT) platform and its utility in all forms audio-visual and telecommunication in public health care at all times and under pandemic/epidemic situations.

## Conclusion

Hospitals are among the most complex institutions in a community. They are staffed by a multidisciplinary team delivering a multiplicity of health services to a highly diverse patient population generally suffering, collectively, from a wide range of health problems. An epidemic/pandemic requires a health facility to alter its priorities and adapt its work routines to mount a coordinated, systemic response to a rapidly evolving, potentially complex situation. Meeting the national and international guidelines and SOPs under local situations can be challenging. As detailed in the earlier pages, the experiences and the lessons learned reveal that the workforce that manages this terrible pandemic requires the right attitude, transparent communication, clear vision, togetherness, empathy, wise approach in patient care, and follow-up. The learning is never complete. Every day poses new challenges that need to be converted to opportunities to learn more and combat this global pandemic. Any mild decline in the incidence of cases and mortality rate should not create an assumption that the battle against COVID is won. A return of the wave of cases is always anticipated, which signals improved preparedness with the lessons learned over the last few months. The hospital with this experience is now equipped and trained to care for any such similar health emergency or outbreak in the future. The university with its teaching medical facility has gained definitive experience and is poised to join hands with other such health care organizations, to combat this global pandemic with its ability to successfully adapt the guidelines of state and national bodies. Despite various limitations and unexpected challenges, the strong structural reinforcement and cohesive workforce along with the transformation of the hospital being “an effective COVID care center” is a “*story of a journey worth shared*.”

While Indian medical schools were set with curricula as per regulatory bodies, a sudden hairpin turn to embrace molecular diagnostics was more than challenging in terms of infrastructure and expertise at all levels. For institutions, such as MGMCRI, a hairpin turn never came because the institution had a well-established Inter-disciplinary Research & Diagnostics Center (CIDRF). Therefore, the inclusion of COVID diagnostics was only policy preparedness. On the other hand, in institutions where medical education was the only primary responsibility, the hairpin turn was a difficult makeover to manage in time. With this experience, a new theory of learning across disciplines has come into focus that has led to different approaches to the design of molecular diagnostic laboratories, highly qualified personnel to establish a functional competence, and training of the medical fraternity to relay such competence. Additionally, ICMR and NABL have rightly set regulations normalizing such establishments. Equally important, the growth of interdisciplinary approaches and new scientific collaborations have begun to make the path from basic research to pandemic management to educational practice somewhat more visible, if not yet easy to travel.

Finally, we share our real-time ground-level experiences in the hope that the documentation will lead to more pragmatic policymaking and devising standard operating procedures in the future that are particularly tailor-made to countries with limited human, infrastructural, and financial resources and that are behind the curve in the spread of the pandemic.

## Data Availability Statement

The original contributions presented in the study are included in the article/supplementary material, further inquiries can be directed to the corresponding author/s.

## Author Contributions

VC, SP, RP, LS, HK, AB, BSi, RK, KR, SP, and KA: Hospital Core COVID Committee. BSu and JE: lab services. PM, AP, and SR: drafting and review of manuscript. SR: initial concept, implementation, and management. All authors contributed to the article and approved the submitted version.

## Conflict of Interest

The authors declare that the research was conducted in the absence of any commercial or financial relationships that could be construed as a potential conflict of interest.
